# Integration of single-cell and bulk RNA sequencing data reveals that CYTOR is a potential prognostic and immunotherapeutic response marker for skin cutaneous melanoma

**DOI:** 10.7150/jca.94823

**Published:** 2024-05-28

**Authors:** Ming Zhang, Yikun Ju, Lei Xue, Xueheng Zhao, Xuezheng Xu, Geng Wu, Hao Bo, Zailong Qin

**Affiliations:** 1 The First People's Hospital of Lianyungang, The First Affiliated Hospital of Kangda College of Nanjing Medical University, The Affiliated Lianyungang Hospital of Xuzhou Medical University, Lianyungang, 220005, China.; 2 Department of Burn and Plastic Surgery, The Second Xiangya Hospital, Central South University, Changsha, 410011, China.; 3 Department of Pathology, Hunan Cancer Hospital, The Affiliated Cancer Hospital of Xiangya School of Medicine, Central South University, Changsha, 410013, China.; 4 NHC Key Laboratory of Human Stem Cell and Reproductive Engineering, Institute of Reproductive and Stem Cell Engineering, School of Basic Medical Science, Central South University, Changsha, 410006, China.; 5 Department of Orthopaedics, Hunan Cancer Hospital and The Affiliated Cancer Hospital of Xiangya School of Medicine, Central South University, Changsha, 410013, China.; 6 Guangxi Key Laboratory of Reproductive Health and Birth Defect Prevention, Guangxi Key Laboratory of Precision Medicine for Genetic Diseases, Guangxi Key Laboratory of Birth Defects and Stem Cell Biobank, Genetic and Metabolic Central Laboratory, Guangxi Clinical Research Center for Pediatric Diseases, Maternal and Child Health Hospital of Guangxi Zhuang Autonomous Region, Nanning, 530003, China.

**Keywords:** CYTOR, SKCM, immunotherapeutic, immune response, lncRNA

## Abstract

Skin cutaneous melanoma (SKCM) is a highly malignant tumor that is prone to immune escape and distant metastasis. Immunotherapy is considered to be the best treatment for patients with SKCM. However, not all patients benefit from it. We observed a significant differential expression of the lncRNA CYTOR in patients with SKCM based on single-cell and bulk RNA sequencing data mining results. The results showed that compared to normal tissue lncRNA CYTOR expression was significantly upregulated in SKCM tissue. Subsequently, we validated this finding in clinical samples, and we also found that the expression of lncRNA CYTOR in SKCM was higher as it progressed. lncRNA CYTOR was differentially expressed in patients who responded to immunotherapy, suggesting that it may serve as a biomarker to predict the efficacy of SKCM immunotherapy. In-depth analysis revealed that lncRNA CYTOR expression was strongly correlated with immune cell infiltration, immune response, and immune checkpoint expression. Meanwhile, our experiments revealed that CYTOR affects SKCM cell invasion and clone formation and is associated with the activation of the EMT pathway. In summary, our findings illustrate, for the first time, the value of CYTOR as a potential prognostic and immunotherapeutic response marker in SKCM.

## Introduction

Skin cutaneous melanoma (SKCM) is a class of skin tumors with high rates of metastasis and lethality 1. Although the early cure rates for SKCM surgery are currently high, currently, the survival and prognosis of patients with mid- to late-stage melanoma remain poor 2, 3. During tumor development, cells continue to generate new mutations, which result in the formation of many subpopulations of cells that differ in their immune characteristics, growth rate, and metastatic ability. This explains the different clinical responses of each subpopulation to drugs or radiation therapy 1. Advanced melanoma is insensitive to conventional radiotherapy and chemotherapy but prone to distant metastases 2. With extensive investigation conducted on the human immune system, it is evident that the body's immune defense system can counter various malignancies 4. Immunotherapy has been studied for more than 100 years, and immunotherapy for malignant melanoma is one of the key areas of research 4. While immune checkpoint therapy is useful for SKCM, many patients do not respond to the treatment 4-6. Therefore, identifying appropriate prognostic markers related to immunotherapeutic response is valuable for patient stratification and individualized treatment 3.

As genomics continues to evolve, researchers are increasingly recognizing the critical role of non-coding RNAs, which were previously considered to be non-functional gene fragments 7. Among them, multiple non-coding RNAs are long-stranded non-coding RNAs (lncRNAs). They have been shown to play an important role in the progression of tumors 7, 8. Several lncRNAs have been shown to be differentially expressed in melanomas, and their expression has been shown to correlate with the metastasis and prognosis of melanomas 9-11. Du *et al.* 12 identified LINC02249 is a prognostic biomarker for cutaneous melanoma and associated with an immunosuppressive microenvironment. Zhang *et al.* 13 analyzed the potential value of lncRNA-PRRT3-AS1 as a therapeutic target in SKCM. This suggests that lncRNAs are valuable as biomarkers. However, the function and clinical value of most lncRNAs in melanomas are yet to be elucidated.

Single-cell sequencing technology, which can be used to obtain genetic information from individual cells, allows a more detailed study of malignant tumors 14. In recent years, it has provided a deeper understanding of various types of tumors, including melanomas, at the cellular biological level 14. Biermann *et al.* 15 used single-cell sequencing to establish a multi-omics single-cell atlas of melanoma brain metastases. It provides important insights for studying tumor microenvironmental characteristics and genome of brain metastases. Li *et al.* 16 used single-cell RNA sequencing (scRNA-seq) to map the transcriptional profile of limbic melanoma, providing a basis for subsequent studies to explore suitable immunotherapeutic targets.

In this study, using relevant data based on single-cell RNA sequencing technology, we found that lncRNA CYTOR play an important role in SKCM, and their relevance in immune infiltration and immune response makes them potential biomarkers for predicting the efficacy of immunotherapy. Our findings provide a valuable resource for us to fully explore the information stored in lncRNAs. In addition, we investigated its function *in vitro*, and these data inform the precise treatment and prognosis of SKCM patients.

## Materials and methods

### Data collection and analysis

The Smart-seq2 single-cell transcriptome sequencing dataset SKCM_GSE115978_aPD1 was acquired from Tumor Immune Single-cell Hub 2 (TISCH2) (http://tisch.comp-genomics.org/home/) 17 and re-clustered using the Uniform Manifold Approximation and Projection (UMAP) algorithm for dimensionality reduction. Following this, it was subjected to re-clustering analysis using the Seurat package from R. The differentiation potential of different single-cell subpopulations was assessed using CytoTRACE (Download the CytoTRACE R package v0.3.3 using the following link: https://cytotrace.stanford.edu/CytoTRACE_0.3.3. (tar.gz)).

### Data mining from databases

High-throughput RNA-Seq mapped transcriptional data per kilobase per million reads were obtained from the TCGA (https://portal.gdc.cancer.gov/; version 1.28.0) SKCM project. The log2 transformation was then performed for correlation analysis. Boxed scatter plots of variance expressions were drawn using the GEPIA2 online tool (http://gepia2.cancer-pku.cn/#index) 18. To further validate our results, the gene expression datasets GSE22153, GSE22154, GSE19234, GSE19293, GSE65904, and GSE99898 were obtained using the BEST online tool (https://rookieutopia.com/app_direct/BEST/). The detailed clinical parameters of the GSE19234 19, GSE19293 20, GSE22153 21, GSE65904 22, and GSE115978 23 datasets were in **Supplementary table 1**. GSEA, CYTOR, SKCM immune correlation analysis, and analysis of drug sensitivity of patients were also performed using the BEST online tool, based on default parameters. The analysis of data related to CYTOR expression in circulating tumor cells was performed using the ctcRbase online database (http://www.origin-gene.cn/database/ctcRbase/.) 24. The methylation data of CYTOR in tumor and normal tissues was retrieved from the DiseaseMeth version 2.0 database (http://bio-bigdata.hrbmu.edu.cn/diseasemeth/.) 25.

### Clinical sample collection and processing

Melanoma tissue samples were obtained from Hunan Cancer Hospital of Xiangya School of Medicine, with 20 cases of melanoma and 5 cases of normal tissues **(Supplementary table 2)**. All patients had a confirmed diagnosis of SKCM without surgery or other treatment and were free of underlying diseases such as diabetes mellitus, hypertension, and coronary artery disease. The fresh tissues were stored in liquid nitrogen. Following this, total RNA from the tissues was extracted for correlation verification. This study was approved by the Ethics Committee of Hunan Cancer Hospital of Xiangya School of Medicine, Central South University.

### Cell culture and siRNA transfection

A875 and A375 cell lines were purchased from Procell company (Wuhan, China). Cells were cultured in a cell culture incubator at 37 ℃, 5% CO_2_, and 95% humidity. When the cell density was 70%-90%, the cells were either passaged or frozen and stored. We transfected the siRNAs into cells using Lipofectamine 3000. After 48 h of transfection, relevant cell function assays were performed along with RNA extraction. After 72 h of transfection, cell proteins were harvested and western blotting was performed. The siRNAs purchased from RiboBio (Guangzhou, China) were as follows: CYTOR-siRNA1: UGAUCGAAUAUGACAGACACCGAAA and CYTOR-siRNA2: UCUAUGUGUCUUAAUCCCUUGUCCU.

### qRT-PCR

After the cells were transfected for 48 h, they were digested using the TRIzol method, and total RNA was extracted. TRIzol was added to the tissue samples, which were then ground using magnetic beads, using the method used for cellular RNA extraction. Following this, the mass and concentration of the obtained RNA were measured using a spectrophotometer. After RNA quantification, the RNA was reverse-transcribed to cDNA using a reverse transcription kit. Real-time fluorescence quantitative PCR (qRT-PCR) was performed using SYBR Green to validate the expression of the target molecule. The sequences of the relevant primers are as follows: LINC00152-forward: 5'-CACTGAAAATCACGACTCC-3'; LINC00152-reverse: 5'-AAATGGGAAACCGACCAGAC-3'.

### Cell invasion analysis using the transwell assay

We performed functional experiments using the cells after siRNA transfection for 48 h. Cells were digested using 0.25% trypsin for 2 min, after which digestion was terminated using complete medium supplemented with FBS (Inner Mongolia Opcel Biotechnology Co. Ltd., China). Cells were washed twice using PBS. The cells were counted using a cell counter. The invasive potential of cells was measured using the transwell assay. A875 and A375 cells (2 × 10^4^ cells/well) were inoculated for 36 h above the 8 mm chamber (the upper chamber was coated with Matrigel in advance) (Corning Inc., NY-Corning, USA) in 200 mL of medium supplemented with 2% FBS and below the chamber, in 600 mL of the medium supplemented with 15% FBS. The wells were stained using 0.1% crystal violet solution, after which images were recorded using a microscope. Five fields of view were selected for counting and analysis.

### Cell clone formation experiment

The cells after transfection for 48 h were collected and counted and inoculated in 6-well cell culture plates, with 500 cells per well. The cell culture medium was changed every 3 days and the cell culture status was observed. Cells were fixed using 4% paraformaldehyde on day 7 or 10, when there were more than 50 cells per cell cluster. The cells were later stained using crystal violet stain and imaged for counting. Three replicate wells were included per group to ensure accuracy.

### Western blotting

Cell precipitates were harvested after transfection for 72 h and used to extract proteins for western blotting. Following this, protein quantification was performed using a bicinchoninic acid protein quantification kit. Next, 30 µg of cellular protein was added to each well containing 4%-20% FuturePAGE gel (ACE Biotechnology, Nanjing, China). After electrophoresis, the proteins were transferred onto polyvinylidene fluoride membranes and blocked using 5% skim milk at room temperature for 1 hour. This was followed by overnight treatment with primary antibodies (1:1000, Vimentin, p-AKT, AKT, P-PI3K, and GAPDH) at 4 °C. The next day, after washing three times with TBST, the membranes were treated for 1 h with a secondary antibody (1:1000, HRP-linked anti-rabbit/anti-mouse IgG) at 37 °C. After washing three times with TBST, images were acquired and analyzed using the Vilber FUSION fx6.uedge imaging system.

### Statistical analysis

Differences between two groups were analyzed using the Student's t-test, and differences between multiple groups were analyzed using ANOVA. Survival curves were compared using the log-rank test. Correlation analysis was performed using the Pearson method. All graphs in this study were created using online tools or GraphPad software. P-values less than 0.05 were considered to be statistically significant.

## Results

### Re-clustering analysis and differential gene screening based on single-cell sequencing data

**Figure 1:**
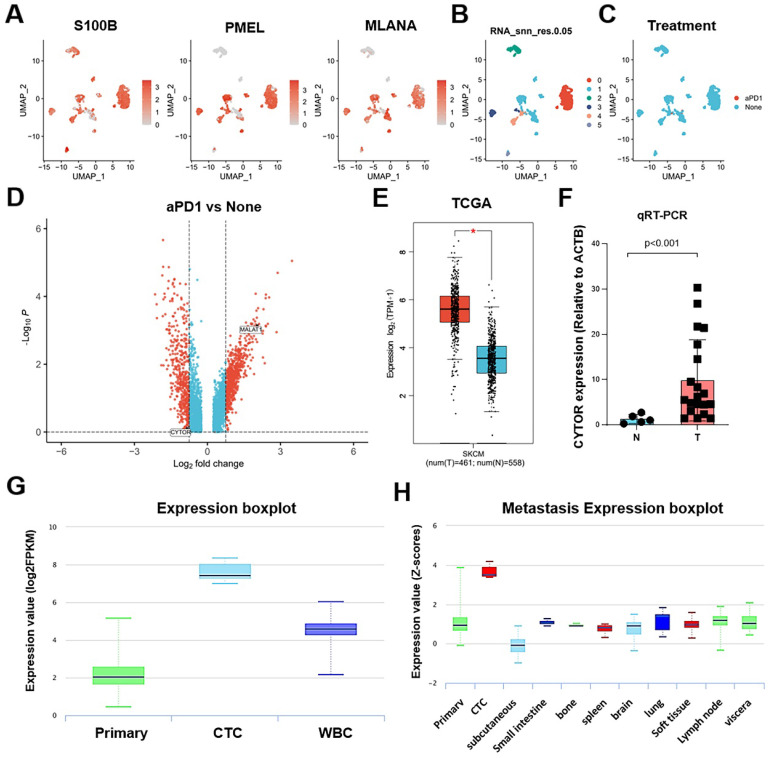
** Re-clustering analysis and differentially expressed gene screening based on transcriptome sequencing data. (A)** Expression of marker genes S100B, PMEL, and MLANA in tumor cells. **(B&C)** Cluster classification of tumor cells.** (D)** Differential gene expression in patients who respond and do not respond to anti-PD-1 therapy.** (E)** Differential expression of CYTOR in tumor and normal tissues based on TCGA-SKCM data.** (F)** CYTOR expression in clinical samples.** (G&H)** CYTOR expression in primary sites and circulating tumor cells. *:P<0.05, **:P<0.01.

We used the UMAP algorithm to re-cluster SKCM tumor cells from the SKCM_GSE115978_aPD1 dataset obtained from TISCH2 after dimensionality reduction. The marker genes S100B, PMEL, and MLANA showed significantly high expression in tumor cells in the SKCM_GSE115978_aPD1 dataset **(Figure 1A)**. Further, we clustered the tumor cells into subclusters and obtained six subclusters **(Figure 1B)**, with subclusters 1 and 4 being treatment response-related subclusters **(Figure 1C)**. Following this, we explored the differentially expressed genes that influenced the response to aPD1 treatment. We extracted relevant data from cells that responded to aPD1 treatment as well as non-responsive cells. After log2 transformation, we mapped the volcano plots of the differentially expressed genes. While there were a few lncRNAs among the differentially expressed genes, CYTOR showed one of the significant differences in expression **(Figure 1D)**. We analyzed the expression of CYTOR in tumor and normal tissues using the TCGA_SKCM dataset (461 tumor samples and 558 normal samples). CYTOR showed a significantly higher expression in tumor samples than in normal tissues (P<0.05)** (Figure 1E)**. We repeated the validation using previously collected clinical samples (24 tumor samples and eight normal samples). After RNA extraction from the tissue samples, qRT-PCR analysis was performed showing that CYTOR expression was significantly higher in tumor tissues (P<0.01) **(Figure 1F)**. Thus, CYTOR may exhibit high differential expression in SKCM. We further analyzed the expression of CYTOR in different tissues and cells using the ctcRbase online database. Surprisingly, CYTOR showed a significantly high expression in circulating tumor cells **(Figure 1G and 1H)**. Thus, CYTOR may exhibit high differential expression in SKCM, especially in circulating tumor cells, and may be used for the diagnosis of SKCM.

### Clinical significance of CYTOR expression in SKCM

**Figure 2:**
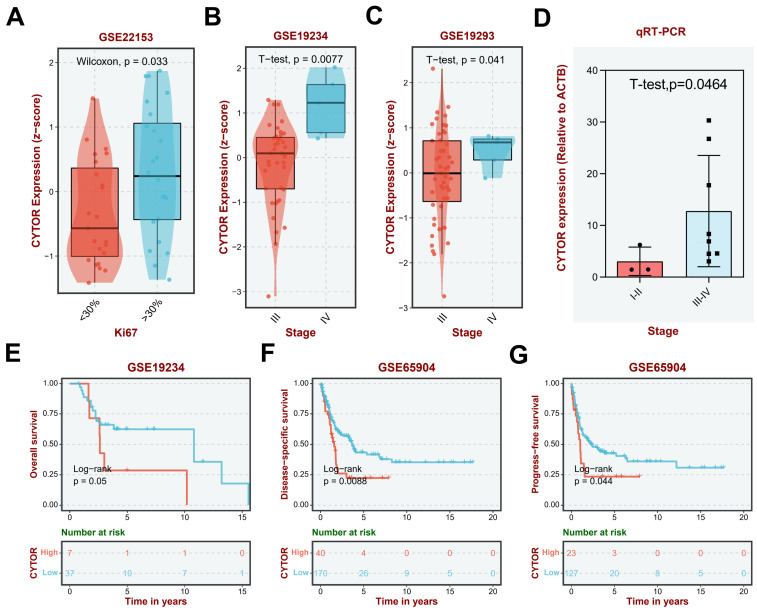
** Clinical significance of CYTOR expression in SKCM. (A)** Correlation of CYTOR expression with Ki67 positivity.** (B&C)** Correlation of CYTOR expression with the staging of patients with SKCM. **(D)** Correlation of CYTOR expression with SKCM patient staging validated using clinical samples. **(E, F, G)** Correlation of CYTOR expression with overall survival, disease-specific survival, and progression-free survival in patients with SKCM.

We further analyzed the clinical significance of CYTOR in SKCM based on findings from different datasets. Clinically, Ki67 is primarily used to label cells in the proliferation cycle. A higher rate of Ki67 labeling indicates more rapid tumor growth, poorer tissue differentiation, and poorer relative prognosis. Our analysis revealed higher and significantly different CYTOR expression in the group with >30% Ki67 labeling compared with that in the group with <30% Ki67 labeling (Wilcoxon, p=0.033), based on the GSE22153 dataset **(Figure 2A)**. Additionally, the expression of CYTOR appeared to be higher in patients with mutant (Mut) SKCM than with wild-type (WT) SKCM (T-test. P=0.035)** (Supplementary Figure 1)**. We speculated that high CYTOR expression may predict a poorer prognosis for patients with SKCM. We then analyzed the expression of CYTOR in SKCM patients at different stages. Consistent with previous speculations, patients with more advanced stages of SKCM had higher CYTOR expression. CYTOR expression was significantly higher in patients with stage IV SKCM than in patients with stage III SKCM, based on GSE19234 (*t*-test, P=0.0077) and GSE19293 (T-test. P=0.041)** (Figure 2B, C)**. We collated and analyzed data from previously collected clinical samples, re-comparing CYTOR expression in patients at different disease stages. Consistent with the findings of the dataset-based analysis, CYTOR expression was significantly higher in patients with stage III-IV SKCM than in patients with stage I-II SKCM (T-test, P=0.0464) **(Figure 2D)**. To further analyze the effect of CYTOR expression on the prognosis of SKCM, SKCM patients were divided into high-risk and low-risk groups according to the expression level of CYTOR. Data between groups were analyzed using Kaplan-Meier survival analysis. Overall survival was shorter in the high-risk group than in the low-risk group, based on GSE19234 (log-rank, P=0.05)** (Figure 2E)**, GSE99898 (log-rank, P=0.014), and GSE22154 (log-rank, P=0.0019)** (Supplementary Figure 1)** data. Disease-specific survival (log-rank, P=0.0088) **(Figure 2F)** and progression-free survival (log-rank, P=0.044)** (Figure 2G)** were similarly shorter in the high-risk group, based on GSE65904 data. CYTOR expression was higher in patients with advanced SKCM, and the prognosis of patients with abnormally high CYTOR expression also tended to be poorer.

### CYTOR affects cell invasion and progression in SKCM

**Figure 3:**
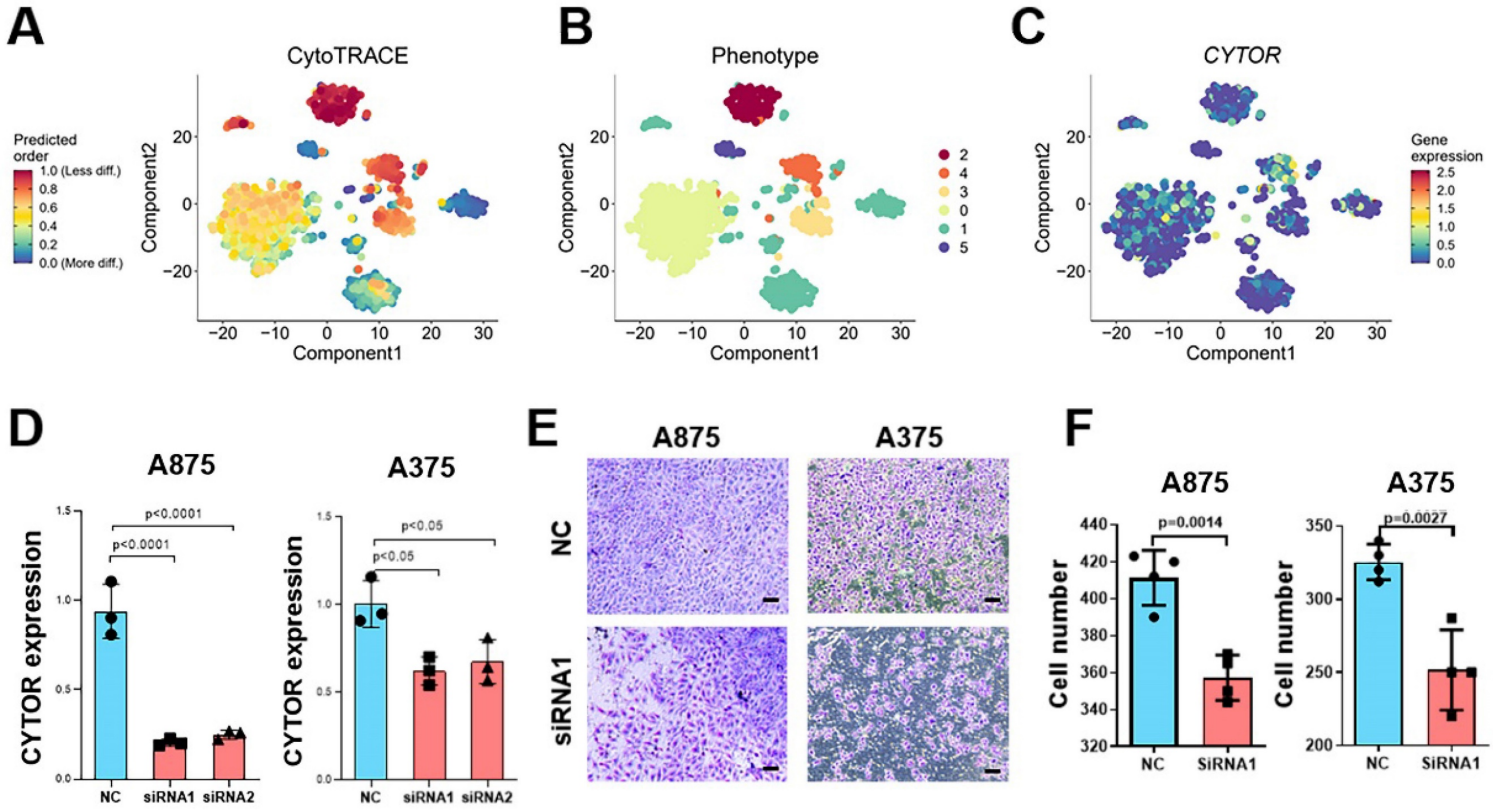
** CYTOR affects cell invasion and SKCM progression. (A, B, C)** Cellular (Cyto) Trajectory Reconstruction Analysis using Gene Counting and Expression (CytoTRACE) was used to perform the proposed time-series analysis of previously acquired single-cell data for predicting the relative differentiation status of cells in the data. **(D)** qRT-PCR for validating the efficiency of CYTOR gene silencing in A375 and A875 cells. **(E & F)** Decreased invasive potential of cells after CYTOR silencing. *:P<0.05, **:P<0.01.

To explore the critical role played by CYTOR in SKCM, we used Cellular (Cyto) Trajectory Reconstruction Analysis using Gene Counting and Expression (CytoTRACE) to perform a proposed time-series analysis of single-cell data for predicting the relative differentiation status of cells. We divided the cells into different groups based on the different degrees of differentiation. CYTOR was not expressed in hypo- or hyper-differentiated cells; rather, it was primarily expressed in moderately differentiated cells. This suggests that CYTOR may play a role in the progression of SKCM **(Figure 3A-C)**. We performed *in vitro* experiments using cell lines of SKCM for exploring their potential role in SKCM progression. We selected A875 and A375 cells to analyze the results of cell function-related experiments. First, two siRNAs targeting CYTOR were designed and the cells were transfected using Lipofectamine 3000. Following this, the transfection efficiency was verified using qRT-PCR. CYTOR expression was significantly lowered in cells after 48h of transfection **(Figure 3D)**. Subsequently, we verified the invasive potential of the cells using the transwell assay. The results showed that the invasive potential of both groups of SKCM cells was significantly inhibited upon CYTOR knockdown **(Figure 3E)**. We also observed that the ability for clone formation was inhibited in A875 cells after transfection (P=0.05) **(Supplementary Figure 2)**.

### Enrichment analysis of the related functions of CYTOR in SKCM

**Figure 4:**
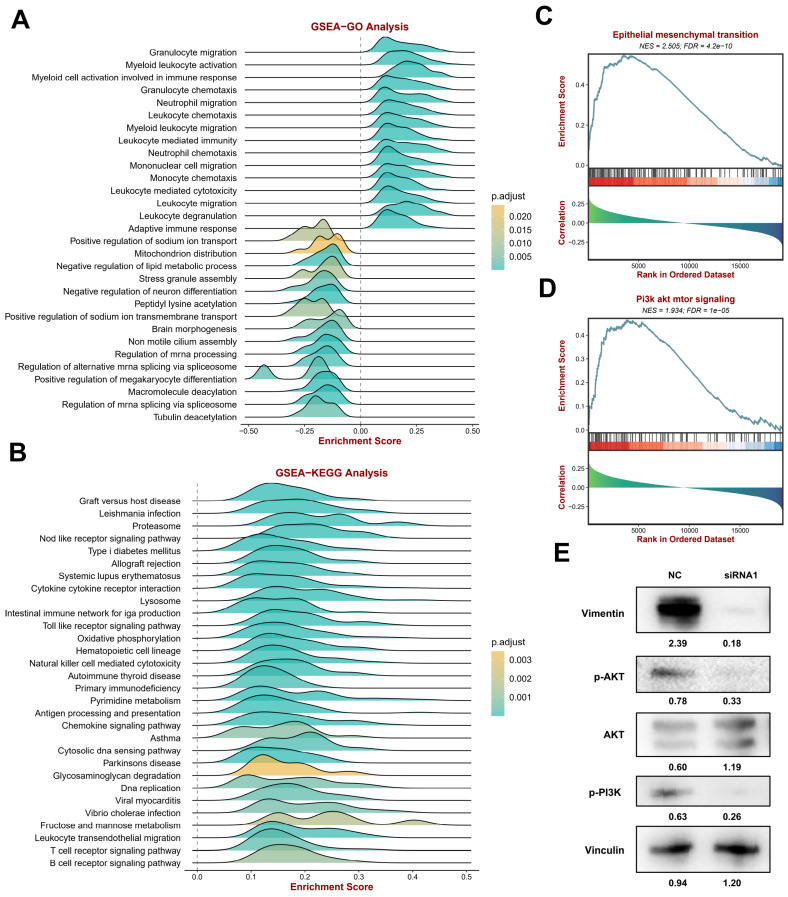
** Enrichment analysis of the functions associated with CYTOR expression in SKCM. (A)** GSEA-GO enrichment analysis. **(B)** GSEA-KEGG enrichment analyses. **(C)** Correlation of epithelial-mesenchymal transition (EMT) with CYTOR expression.** (D)** Correlation of Pi3k akt mtor signaling with CYTOR expression. **(E)** Western blotting results of related molecules after CYTOR knockdown.

To explore CYTOR-related functions and signaling pathways, we divided the TCGA SKCM cohort data into two groups based on CYTOR expression data using the BEST online tool for GSEA. GO enrichment analysis **(Figure 4A, Supplementary table 3)** revealed that many immune-related signals were enriched, such as “Granulocyte migration,” “Myeloid leukocyte activation,” “Myeloid cell activation involved in immune response,” “Granulocyte chemotaxis,” “Neutrophil migration,” “Leukocyte chemotaxis,” “Myeloid leukocyte migration,” “Leukocyte mediated immunity,” “Neutrophil chemotaxis,” “Mononuclear cell migration,” “Monocyte chemotaxis,” “Leukocyte mediated cytotoxicity,” “Leukocyte migration,” “Leukocyte degranulation,” and “Adaptive immune response”. Likewise, many immune-related functions were found to be enriched in the KEGG enrichment analyses, including “Natural killer cell mediated cytotoxicity,” “Antigen processing and presentation,” “Chemokine signaling pathway,” “B cell receptor signaling pathway,” and “T cell receptor signaling pathway” **(Figure 4B, Supplementary table 4)**. The results of the enrichment analysis indicated a strong correlation between CYTOR expression and the immune response. Following this, we analyzed the correlation of epithelial-mesenchymal transition (EMT) and Pi3k akt mtor signaling with CYTOR expression **(Figure 4C, D, Supplementary table 5)**. These two signaling pathways are important for the development and progression of malignant tumors. The two signaling pathways were significantly enriched in the samples with high CYTOR expression. We examined the relevant molecules in SKCM cells after transfection with siRNA using western blotting. Vimentin, P-AKT, and P-Pi3k were downregulated, whereas AKT was upregulated **(Figure 4E)**. We hypothesized that CYTOR plays an important role in the immune response and, in addition, influences SKCM progression through EMT and Pi3k akt mtor signaling.

### CYTOR expression is highly correlated with immune infiltration

**Figure 5:**
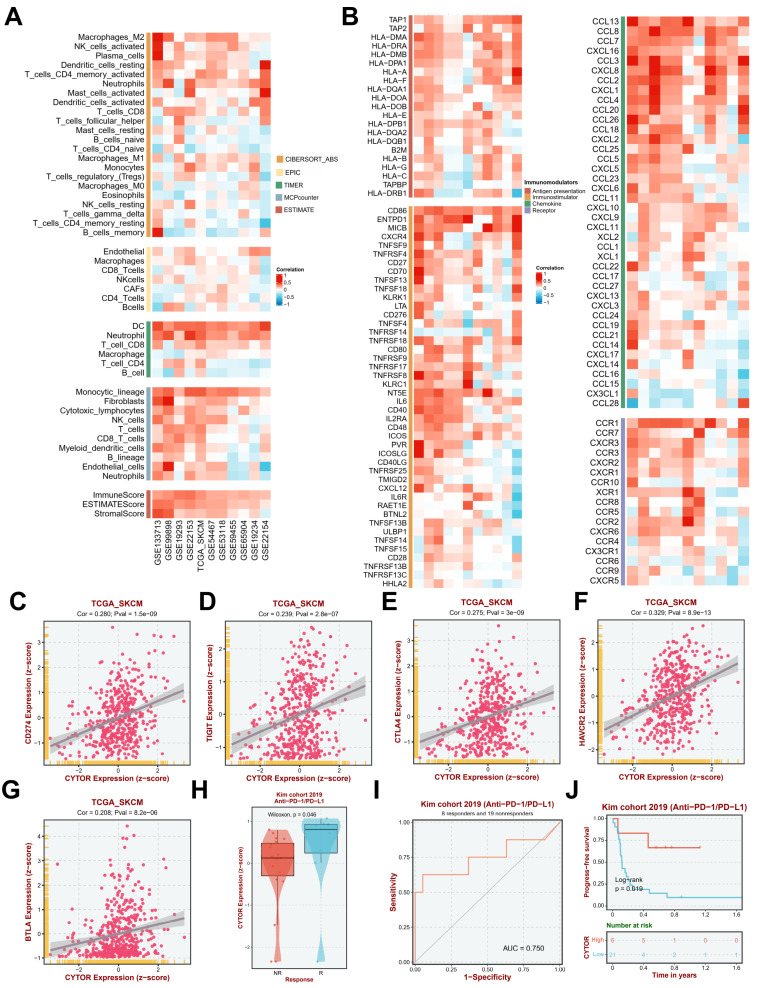
** CYTOR expression is highly correlated with immune infiltration (A)** Correlation between CYTOR expression and immune cell infiltration. **(B)** Correlation between CYTOR expression and immune checkpoint expression.** (C, D, E, F, G)** Correlation between CYTOR expression and CD274, TIGIT, CTLA4, HAVCR2, and BTLA expression. **(H)** CYTOR expression in the anti-PD-1/PD-L1 treatment response cohort. **(I)** Receiver operating characteristic curve of the anti-PD-1/PD-L1 treatment response cohort based on CYTOR expression mapping. **(J)** Kaplan-Meier survival analysis for predicting prognosis in the anti-PD-1/PD-L1 treatment response cohorts.

Since the results of the previous gene enrichment analysis suggested a high correlation between CYTOR expression and immune activity, we further analyzed the immune infiltration of CYTOR in multiple datasets. We performed immune infiltration analysis on multiple datasets (GSE133713, GSE99898, GSE19293, GSE22153, TCGA_SKCM, GSE54467, GSE53118, GSE59455, GSE65904, GSE19234, and GSE22154) based on TIMER (Tumor Immune Estimation Resource), MCPcounter, and ESTIMATE algorithms. CYTOR expression was found to be positively correlated with the infiltration of various types of immune cells, such as dendritic cells (DCs), neutrophils, CD8+ T cells, Monocytic_lineage, and fibroblasts. Moreover, the ImmuneScore, ESTIMATScore, and StromalScore also showed a positive correlation with CYTOR expression in each dataset **(Figure 5A)**. Further analysis revealed that in addition to immune cell infiltration, CYTOR expression was positively correlated with the expression of various immune-related cytokines, receptors, and chemokines, such as CD86, EMTPD1, IL-6, CCL13, CCL8, CCL7, CXCL16, and CCL3, among others **(Figure 5B)**. We focused on the correlation among CD274, TIGIT, CTLA4, HAVCR2, BTLA, and CYTOR expression based on information from the TCGA_SKCM dataset. All the genes showed strong positive correlation (CD274, Cor=0.280, Pval=1.5e-09; TIGIT, Cor=0.239, Pval=2.8e-07; CTLA4, Cor=0.275, Pval=3e-09; HAVCR2, Cor=0.329, Pval=8.9e-13; BTLA, Cor=0.208, Pval=8.2e-06) **(Figure 5C-G)**. This suggests that CYTOR plays an important role in immune activity in SKCM. We analyzed CYTOR expression in patients who underwent Anti-PD-1/PD-L1 treatment based on data from the Kim cohort 2019 and observed higher CYTOR expression in patients who responded to Anti-PD-1/PD-L1 treatment compared with those who did not (Wilcoxon, P=0.046) **(Figure 5H)**. We performed receiver operating characteristic (ROC) curve analysis on this cohort (AUC=0.750). Our results indicated that CYTOR expression has good predictive value and may be a potential immunotherapeutic target **(Figure 5I)**. In contrast, Kaplan-Meier survival analysis revealed that patients with high CYTOR expression had better prognostic value in patients who underwent anti-PD-1/PD-L1 treatment (Log-rank, P=0.019) **(Figure 5J)**. Collectively, CYTOR expression is highly correlated with immune activity. It may serve as a predictor of immunotherapeutic response as well as a potential target for immunotherapy.

### Analysis of the sensitivity of high CYTOR expression to antitumor drugs

**Figure 6:**
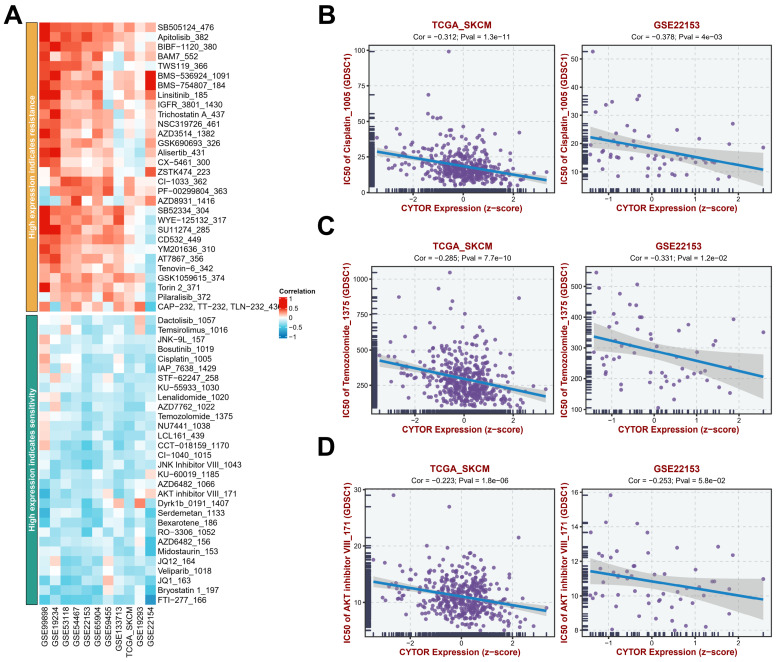
** Analysis of antitumor drug sensitivity in cases with high CYTOR expression. (A)** Antitumor drug sensitivity profiling in multiple datasets.** (B, C, D)** Correlation between CYTOR expression and the IC_50_ of antitumor drugs (cisplatin, temozolomide, and AKT inhibitor).

To further analyze the potential value of CYTOR expression in SKCM treatment, we analyzed the antitumor drug sensitivity of patients with SKCM with high CYTOR expression based on findings from multiple datasets (GSE99898, GSE19234, GSE53118, GSE54467, GSE22153, GSE65904, GSE59455, GSE133713, TCGA_SKCM, GSE19293, GSE22154 **(Figure 6A, Supplementary table 6)**. Patients with SKCM with high CYTOR expression showed resistance to some antitumor drugs, such as SB-505124, Apitolisib_382, BAM7_552, BMS-536924_1091, and BMS-754807_184, and sensitivity to FTI-277_166, Bryostatin 1_197, and others. We performed an in-depth analysis of the correlation between CYTOR expression and the efficacy of several common antitumor drugs based on data from the TCGA_SKCM and GSE22153 datasets. The semi-inhibitory concentrations (IC_50_) of cisplatin, temozolomide, and AKT inhibitor decreased gradually with the increase in CYTOR expression, exhibiting a negative correlation **(Figure 6B-D)**. CYTOR expression may be used as a dosing guidance for patients with SKCM to develop personalized and precise treatment plans.

### Methylation analysis of CYTOR

**Figure 7:**
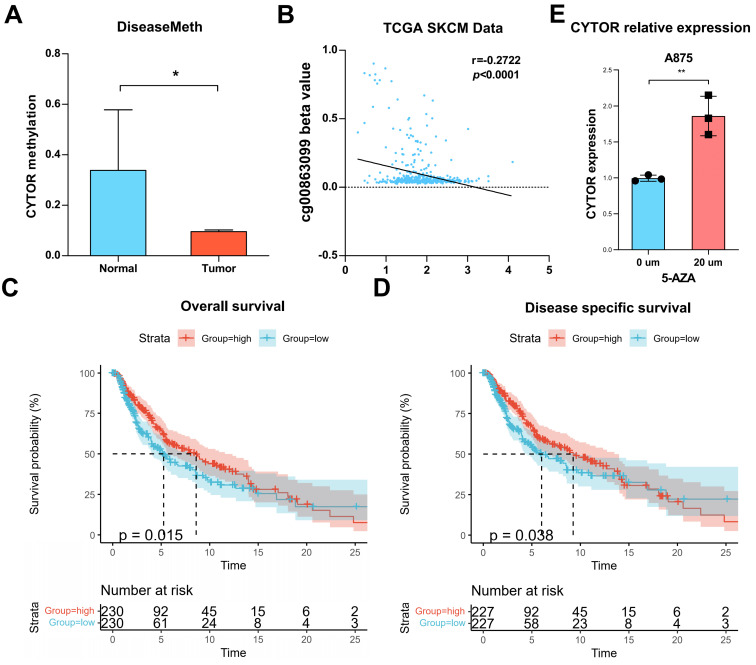
** Analysis of CYTOR methylation. (A)** CYTOR methylation in normal and tumor tissues. **(B)** The correlation between CYTOR-related CpG sites (cg00863099) and CYTOR expression. **(C & D)** CYTOR methylation levels were used to predict the duration of overall survival and disease-specific survival in patients with SKCM.** (E)** CYTOR expression in SKCM cells after treatment with 5-AZA. *:P<0.05, **:P<0.01.

We analyzed the methylation level of CYTOR based on DiseaseMeth. The result revealed that the methylation level of CYTOR was significantly higher in normal tissues than in tumor tissues (P<0.05) **(Figure 7A)**. DiseaseMeth is a database of abnormal methylation focused on human diseases that can be used to analyze the relationship between the methylation levels of genes and various diseases. We evaluated the correlation of the CYTOR-related CpG site (cg00863099) with CYTOR expression based on the TCGA_SKCM dataset. The results showed a negative correlation (r=-0.2722, P<0.0001) **(Figure 7B)**. Further, we evaluated the effect of CYTOR methylation on the prognosis of patients with SKCM using Kaplan-Meier survival analysis. The analysis revealed that the lower the CYTOR methylation level, the lower the overall survival (p=0.015) and disease-specific survival of patients (p=0.038) **(Figure 7C, D)**. 5-Azacytidine nucleoside (5-AZA) is a hypomethylating and potent growth-inhibiting and cytotoxic agent that inhibits DNA methyltransferase. We treated A875 cells with 20 µm 5-AZA for 48 h, after which we measured CYTOR expression using qRT-PCR. We found that CYTOR expression was upregulated in drug-treated cells (p<0.01) **(Figure 7E)**. In conclusion, the methylation level of CYTOR was correlated with the survival of patients with SKCM.

## Discussion

CYTOR (LINC00152) is a critical lncRNA considered to play an important role in tumorigenesis and development 26-28. CYTOR has been identified as an oncogene in various cancers, including gastric, hepatocellular, colon, gallbladder, and renal cell cancers 26-29. Zhao *et al.* 30 found that CYTOR expression is involved in gastric cancer cell cycle arrest, apoptosis, EMT, cell migration, and invasion. Cai *et al.* 31 found that CYTOR promotes gallbladder cancer metastasis and EMT through miR-138-regulated HIF-1α. Zhang *et al.* 32 systematically reviewed the value of CYTOR as a predictor of lymph node metastasis and survival in human cancers and performed a meta-analysis. CYTOR molecules play an important role in various tumors, but the function of CYTOR in SKCM is yet to be reported 29. We analyzed the expression of CYTOR based on evidence from the TCGA database and showed that CYTOR was expressed at high levels in patients with SKCM. We validated the data using qRT-PCR, and our findings were consistent with the predicted results. Specifically, our analysis based on a set of single-cell data revealed that the CYTOR expression profiles of patients responding to anti-PD-1 therapy were significantly different from those of non-responding patients. The above results suggest that CYTOR expression may be important in SKCM.

Further, we analyzed CYTOR expression based on the single-cell sequencing dataset. CYTOR was found to be expressed at high levels in circulating tumor cells. Thus, CYTOR may be used for the liquid biopsy of SKCM to facilitate the early diagnosis of tumors. Additionally, when we analyzed the clinical characteristics of patients with SKCM, high CYTOR expression predicted a worse prognosis for patients with SKCM. Unlike other studies, we also identified for the first time a potential regulatory role of CYTOR methylation and the use of CYTOR methylation level as an effective marker.

Immune checkpoint inhibitors (ICIs) play an important role in the treatment of patients with melanoma, but some patients exhibit tolerance to ICIs 23. Jerby-Arnon *et al.* 23 explored the state of malignant melanoma cells that promotes immune evasion using single-cell sequencing technology and predicted the clinical response to anti-PD-1 therapy in an independent cohort of 112 patients with melanoma. We used bioinformatics analysis based on this dataset to explore the relevant lncRNAs affecting the outcomes of SKCM immunotherapy. We first identified that CYTOR expression is associated with the immunotherapy response of SKCM. Anti-PD-1 therapy is a classic therapeutic strategy, and CYTOR expression differs significantly in patients who respond or do not respond to anti-PD-1 therapy. The results of gene enrichment analysis showed that CYTOR expression was associated with numerous immune-related functions, such as “Natural killer cell mediated cytotoxicity,” “Antigen processing and presentation,” “Chemokine signaling pathway,” “B cell receptor signaling pathway,” and “T cell receptor signaling pathway,” among others. More importantly, the expression molecule has a high correlation with the abundance of numerous immune cells and immune checkpoints, which is of great significance for further research on immunotherapeutic targets against malignant melanoma. DCs are the most powerful antigen-presenting cells and play an important role in tumor immunity. CYTOR expression was found to be significantly and positively correlated with the infiltration of DC cells. In addition, the infiltration of numerous immune cells associated with the tumor immune microenvironment, such as neutrophils, monocytes, and fibroblasts, among others, is strongly correlated with CYTOR expression. Meanwhile, the expression of numerous immune checkpoints, such as CCL13, CCL8, CCL7, CCL4, and CCR1, was positively correlated with CYTOR expression. We also specifically analyzed the correlation between CYTOR expression and the sensitivity to various antitumor drugs. CYTOR expression showed a negative correlation with the sensitivity to some commonly used antitumor drugs, such as cisplatin and temozolomide, among others. In summary, CYTOR plays a very important role in the immunity of tumors. It is also expected to be useful for predicting the prognosis of immunotherapy.

**Figure 8:**
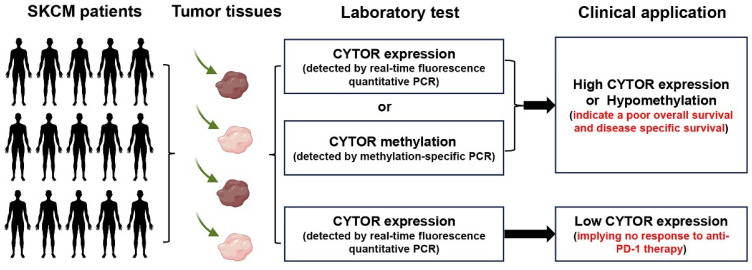
** Schematic representation of the clinical translation of CYTOR.** The expression of CYTOR and its methylation in SKCM tumor tissues was analyzed by PCR and used to predict the prognosis and immunotherapy response of SCKM patients.

In conclusion, our findings illustrate the important role and value of CYTOR in SKCM. The findings further expand on the roles played by CYTOR in malignancies and may improve the use of CYTOR in tumor diagnosis and treatment **(Figure 8)**. In particular, we were surprised to observe a high correlation between CYTOR expression and immune cell infiltration, immune molecule expression, and immunotherapeutic responses. The expression of CYTOR and its methylation in SKCM tumor tissues was analyzed by qRT-PCR and used to predict the prognosis and immunotherapy response of SCKM patients. CYTOR is a potential target for predicting the sensitivity to immunotherapy in patients with SKCM and a potential marker for predicting the immunotherapeutic response.

## Supplementary Material

Supplementary figures.

Supplementary table 1.

Supplementary table 2.

Supplementary table 3.

Supplementary table 4.

Supplementary table 5.

Supplementary table 6.

## References

[1] Strashilov S, Yordanov A (2021). Aetiology and pathogenesis of cutaneous melanoma: current concepts and advances. Int. J. Mol. Sci.

[2] Skudalski L, Waldman R, Kerr PE, Grant-Kels JM (2022). Melanoma: an update on systemic therapies. J. Am. Acad. Dermatol.

[3] Pavri SN, Clune J, Ariyan S, Narayan D (2016). Malignant melanoma: beyond the basics. Plast. Reconstr. Surg.

[4] Koller KM, Wang W, Schell TD, Cozza EM, Kokolus KM, Neves RI (2016). Malignant melanoma-The cradle of anti-neoplastic immunotherapy. Crit. Rev. Oncol. Hematol.

[5] Ahn A, Rodger EJ, Motwani J, Gimenez G, Stockwell PA, Parry M (2021). Transcriptional reprogramming and constitutive PD-L1 expression in melanoma are associated with dedifferentiation and activation of interferon and tumour necrosis factor signalling pathways. Cancers.

[6] Bhave P, Ahmed T, Lo SN, Shoushtari A, Zaremba A, Versluis JM (2022). Efficacy of anti-PD-1 and ipilimumab alone or in combination in acral melanoma. J. Immunother. Cancer.

[7] Durante G, Comito F, Lambertini M, Broseghini E, Dika E, Ferracin M (2021). Non-coding RNA dysregulation in skin cancers. Essays Biochem.

[8] Garofoli M, Volpicella M, Guida M, Porcelli L, Azzariti A (2020). The role of non-coding rnas as prognostic factor, predictor of drug response or resistance and pharmacological targets, in the cutaneous squamous cell carcinoma. Cancers.

[9] Yu X, Zheng H, Tse G, Chan MT, Wu WK (2018). Long non-coding RNAs in melanoma. Cell Prolif.

[10] Zhou X, Rong R, Xiong S, Song W, Ji D, Xia X (2022). Integrated analysis to reveal potential therapeutic targets and prognostic biomarkers of skin cutaneous melanoma. Front. Immunol.

[11] Zhang J, Liu H, Zhang W, Li Y, Fan Z, Jiang H (2020). Identification of lncRNA-mRNA regulatory module to explore the pathogenesis and prognosis of melanoma. Front. Cell Dev. Biol.

[12] Du M, Han L, Shen P, Wu D, Tu S (2022). Long noncoding RNA LINC02249 is a prognostic biomarker and correlates with immunosuppressive microenvironment in skin cutaneous melanoma. J. Oncol.

[13] Zhang W, Xie X, Huang Z, Zhong X, Liu Y, Cheong KL (2022). The integration of single-cell sequencing, TCGA, and GEO data analysis revealed that PRRT3-AS1 is a biomarker and therapeutic target of SKCM. Front. Immunol.

[14] Ziegenhain C, Vieth B, Parekh S, Reinius B, Guillaumet-Adkins A, Smets M (2017). Comparative analysis of single-cell RNA sequencing methods. Mol. cell.

[15] Biermann J, Melms JC, Amin AD, Wang Y, Caprio LA, Karz A (2022). Dissecting the treatment-naive ecosystem of human melanoma brain metastasis. Cell.

[16] Li J, Smalley I, Chen Z, Wu JY, Phadke MS, Teer JK (2022). Single-cell characterization of the cellular landscape of acral melanoma identifies novel targets for immunotherapy. Clin. Cancer Res.

[17] Sun D, Wang J, Han Y, Dong X, Ge J, Zheng R (2021). TISCH: a comprehensive web resource enabling interactive single-cell transcriptome visualization of tumor microenvironment. Nucleic Acids Res.

[18] Tang Z, Kang B, Li C, Chen T, Zhang Z (2019). GEPIA2: an enhanced web server for large-scale expression profiling and interactive analysis. Nucleic Acids Res.

[19] Bogunovic D, O'Neill DW, Belitskaya-Levy I, Vacic V, Yu YL, Adams S (2009). Immune profile and mitotic index of metastatic melanoma lesions enhance clinical staging in predicting patient survival. Proc. Natl. Acad. Sci. U.S.A.

[20] Augustine CK, Jung SH, Sohn I, Yoo JS, Yoshimoto Y, Olson JA Jr (2010). Gene expression signatures as a guide to treatment strategies for in-transit metastatic melanoma. Mol. Cancer Ther.

[21] Jönsson G, Busch C, Knappskog S, Geisler J, Miletic H, Ringnér M (2010). Gene expression profiling-based identification of molecular subtypes in stage IV melanomas with different clinical outcome. Clin. Cancer Res.

[22] Cirenajwis H, Ekedahl H, Lauss M, Harbst K, Carneiro A, Enoksson J (2015). Molecular stratification of metastatic melanoma using gene expression profiling: Prediction of survival outcome and benefit from molecular targeted therapy. Oncotarget.

[23] Jerby-Arnon L, Shah P, Cuoco MS, Rodman C, Su MJ, Melms JC (2018). A cancer cell program promotes T cell exclusion and resistance to checkpoint blockade. Cell.

[24] Zhao L, Wu X, Li T, Luo J, Dong D (2020). ctcRbase: the gene expression database of circulating tumor cells and microemboli. Database.

[25] Xiong Y, Wei Y, Gu Y, Zhang S, Lyu J, Zhang B (2017). DiseaseMeth version 2.0: a major expansion and update of the human disease methylation database. Nucleic Acids Res.

[26] Yu Y, Yang J, Li Q, Xu B, Lian Y, Miao L (2017). LINC00152: A pivotal oncogenic long non-coding RNA in human cancers. Cell Prolif.

[27] Li S, Yao W, Liu R, Gao L, Lu Y, Zhang H (2022). Long non-coding RNA LINC00152 in cancer: Roles, mechanisms, and chemotherapy and radiotherapy resistance. Front Oncol.

[28] Ghafouri-Fard S, Askari A, Hussen BM, Rasul MF, Taheri M, Kiani A (2023). A review on the role of LINC00152 in different disorders. Pathol Res Pract.

[29] Wang H, Liu Y, Tang A (2020). Prognostic Values of Long Noncoding RNA linc00152 in various carcinomas: an updated systematic review and meta-analysis. Oncologist.

[30] Zhao J, Liu Y, Zhang W, Zhou Z, Wu J, Cui P (2015). Long non-coding RNA Linc00152 is involved in cell cycle arrest, apoptosis, epithelial to mesenchymal transition, cell migration and invasion in gastric cancer. Cell Cycle.

[31] Cai Q, Wang Z, Wang S, Weng M, Zhou D, Li C (2017). Long non-coding RNA LINC00152 promotes gallbladder cancer metastasis and epithelial-mesenchymal transition by regulating HIF-1alpha via miR-138. Open Biol.

[32] Zhang J, Yin M, Huang J, Lv Z, Liang S, Miao X (2018). Long noncoding RNA LINC00152 as a novel predictor of lymph node metastasis and survival in human cancer: a systematic review and meta-analysis. Clin Chim Acta.

