# Supplementary material for: Integration of single-cell and bulk RNA sequencing data reveals that CYTOR is a potential prognostic and immunotherapeutic response marker for skin cutaneous melanoma
Source: J Cancer. 2024 May 28;15(12):3890–902. doi: 10.7150/jca.94823 (PMC11190755; doi:10.7150/jca.94823)
Supplement: Supplementary file 1 — Supplementary figures. [file jcav15p3890s1.pdf]

Supplementary material

Integration of single-cell and bulk RNA sequencing data reveals that CYTOR is a potential prognostic and immunotherapeutic response marker for skin cutaneous melanoma

Supplementary Figure1

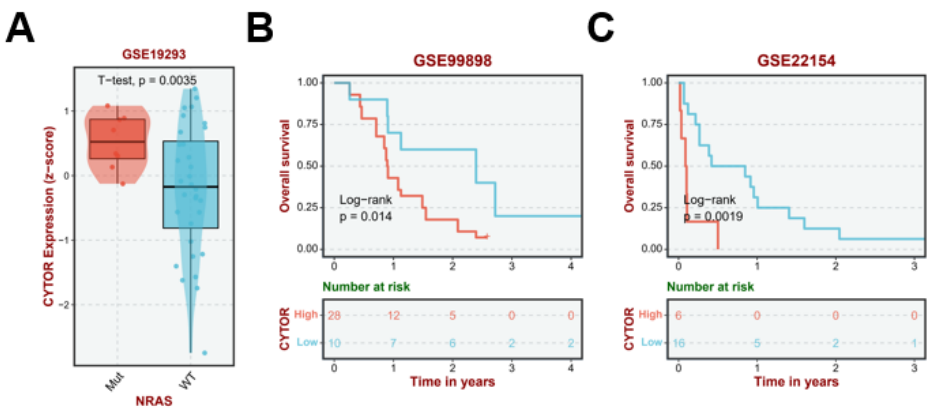

Supplementary Figure 1 (A) Correlation of CYTOR expression with Mut and WT. (B, C) Correlation of CYTOR expression with overall survival in patients with SKCM.

Supplementary Figure 2

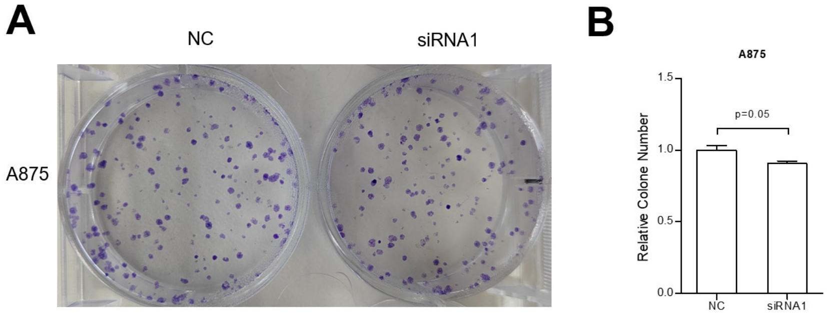

Supplementary Figure 2 (A, B) Decreased cloning ability of cells after CYTOR silencing.
